# Practical Apects of Medical Science

**Published:** 1894-05-26

**Authors:** 


### Practical Aspects of Medical Science.

It is only of late years that we have understood the full significance of the sun worship which is so inherent in our being. Up to now, the beneficial effects of light and of sunshine have been appreciated rather than understood. But the horizon of our knowledge is daily extending regarding the sun's influence on terrestrial affairs, a knowledge which we have acquired through the medium of laborious scientific research. This is pointed out to us in a striking manner by Professor Frankland in an article which he has lately contributed to the *Nineteenth Century*, where he shows us some of the wonders of the great solar power in its control over the phenomena of our earth, a control which dominates despite 93,000,000 miles of intervening space.

Its most recently discovered influence is on micro-organisms the disease-producing powers of which the mighty and distant sun appears to have it in his power to remove. We are, of course, as the Professor reminds us, only on the threshold of this most interesting branch of bacteriological research, but the results which have been already obtained open up the possibility of discoveries of the most vital importance in hygienic science. Microbes, as we all know, whilst they are capable of performing quite indispensable services to man in such important directions as the breaking up of refuse organic matter, the fertilisation of the soil, the production of alcohol, &c., may, and do, become objectionable when they venture outside their particular sphere of utility.

It is in their extreme minuteness that the difficulties lie of keeping them in their proper place; and another difficulty which has to be contended with is the micro-organic fertility. In dealing with the subject one has to bear this fact constantly in mind, the marvellously rapid power of reproduction of these organisms.

Granted fair conditions, it does not take more than twenty or thirty minutes to produce a new generation of bacteria.

Professor Frankland carries on this calculation further. "Thus," without let or hindrance, he reminds us, "a single bacillus would, in the course of twenty-four hours, have given rise to a progeny four times as numerous as the whole population of London, whilst at the end of forty-eight hours their number would be represented by the wholly unmanageable figures—280,000,000,000,000."

But, fortunately, Nature *has* provided many a let and hindrance against the microbe displaying its fullest possibilities in the reproductive line. Many agencies are at work which are not conducive to their fulfilment. These are contained in limited food supplies, adverse circumstances of temperature, and last but not least, the influence of sunshine on micro-organic life. We really owe it to two of our own countrymen that they were the discoverers of the disinfecting properties of solar rays. These two researchers, Blount and Downes by name, first realised this power, in the instance of certain liquids, which, if left in comparative darkness, were capable of undergoing putrefaction, but which, if exposed to the sun's rays, remained untainted. The same liquid invariably exhibited innumerable bacteria when retained in the dark. In this way it was, Professor Frankland tells us, that the lethal action of the sun's rays were shown for the first time, as regards the world of micro-organisms, and quite a new aspect was given to the healthy and inherent craving for light in our houses and general surroundings.

Investigation on this interesting and all-important subject did not by any means end with our two pioneer countrymen. They led the way in the so far unexplored country, but others followed; and whilst all known results point to the same conclusion, their nature has been various and varied.

Since then Dr. Geisler, of St. Petersburg, struck out a new line in extension of this subject. He compared the potency of the sun and the electric light respectively in destroying bacterial life. Resultant on his work, he discovered that whilst an unhappy change was observable in the condition of the typhoid bacillus at the expiration of two or three hours exposure to sunshine, it required three times as long an exposure to the beams of an electric arc lamp of 1,000 candle power, and at the "comparatively" closer quarters of only thirty-nine inches distance.

Professor Frankland gives other instances in illustration of his subject which will go far to convince the sceptical, and within the past few weeks fresh instances have been brought forward which forcibly bear upon the importance of his field of research.

He gives us an epitome of the results of Dr. Palermo's work at Naples dealing with the effects of sunshine on these diminutive organisms. The microbe selected for experiment was Koch's cholera bacillus. This bacillus has proved fatal to guinea pigs after eighteen hours, and is the one generally accredited with the honour of producing Asiatic cholera in man.

Now Dr. Palermo found that after he had, so to speak, "sunned" these microbes, they were quite ineffectual in producing harmful results; those not thus exposed to the sun's light remaining unvaried in their fatal effect on the animals subjected to them.

It would further appear that the same microbes which had been influenced by the "sunning" were not killed or themselves destroyed, "but," as our authority puts it, "their inability to work mischief was due to the removal during this exposure of their virulence or disease-destroying powers." Another matter equally important was further noted in connection with this. These very guinea pigs which, as we are told, survived the above inoculation with the sun-exposed cholera bacilli, were rendered proof against further contracting it, in like manner as vaccination with cow-pox protects against real small-pox. "Thus," as our authority comments, "by exposure to sunshine these disease microbes were not only deprived of their sting, so to speak, but were converted into useful servants in protecting their former victims from the attacks of their still viciously disposed brethren. The study of these minute living organisms," he continues, "which have attracted so much attention during the past twenty years, has also served in this most surprising and unexpected manner to increase our admiration of the marvellous dominion which is thus wielded by the sun; and even if we are not quite prepared to revert to the ancient religion of Khuenaten, and of the Dark Worshippers of Egypt, we shall at any rate deeply respect the material intelligence and beauty of their doctrines, whilst we shall all endorse the poetic imagery of Plato, when he says that 'Truth is the Body of God, and Light is His Shadow.'"

